# Investigation of Structural Phase, Mechanical, and Tribological Characteristics of Layer Gradient Heat-Protective Coatings Obtained by the Detonation Spraying Method

**DOI:** 10.3390/ma17215253

**Published:** 2024-10-29

**Authors:** Dastan Buitkenov, Bauyrzhan Rakhadilov, Aiym Nabioldina, Yerkat Mukazhanov, Meruert Adilkanova, Nurmakhanbet Raisov

**Affiliations:** 1 Research Center Surface Engineering and Tribology, Sarsen Amanzholov East Kazakhstan University, Ust-Kamenogorsk 070000, Kazakhstan; dbuitkenov@vku.edu.kz (D.B.); brahadilov@vku.edu.kz (B.R.); emukazhanov@vku.edu.kz (Y.M.); nraisov@vku.edu.kz (N.R.); 2 International School of Engineering, Daulet Serikbayev East Kazakhstan Technical University, Ust-Kamenogorsk 070002, Kazakhstan; madilkanova@edu.ektu.kz

**Keywords:** detonation spraying, multilayer coatings, thermal barrier coatings (TBCs), phase composition, tribological properties, corrosion resistance, high-temperature oxidation

## Abstract

This paper presents the results of a study of layer gradient thermal protection coatings based on NiCrAlY and YSZ obtained by detonation spraying. Modern gas turbines and high-temperature units operate under extreme temperatures and aggressive environments, which requires effective protection of components from wear, corrosion, and thermal shocks. In this study, the use of layer gradient coatings consisting of alternating layers of NiCrAlY and YSZ was investigated with the aim of solving the problem of thermal stress accumulation due to a smooth change in the composition of the layers. Microstructural and phase analysis showed that alternating layers of NiCrAlY and YSZ formed a dense layer gradient structure with clear interphase boundaries and low porosity. Detonation spraying led to a complete transformation of the monoclinic ZrO_2_ phase into a tetragonal one, which significantly increased the mechanical strength of the coating and its resistance to thermal shocks. Sample 1D1 demonstrated excellent tribological and corrosion properties in a 3.5% NaCl solution, which can be explained by its higher density and reduced number of pores. Mechanical tests revealed stable values of hardness and wear resistance of the coating, especially for the 1D1 coating. Studies have shown that coatings are resistant to thermal shocks, but thicker layers show a tendency to peel off after thermal cycling. The obtained results indicate high prospects for the use of layer gradient coatings based on NiCrAlY and YSZ for the protection of gas turbine components and other high-temperature installations operating under extreme loads and aggressive environments.

## 1. Introduction

Modern gas turbines and other high-temperature installations operate under conditions of extreme temperatures, significant mechanical loads, and aggressive environments [1,2,3,4,5]. These factors require the use of special protective technologies to prevent wear, corrosion, and thermal damage to the metal components. One of the most effective solutions for surface protection is the use of thermal barrier coatings (TBCs), which can increase the reliability and durability of equipment [6,7,8]. Traditional thermal barrier coatings consist of a metallic bonding layer, usually based on MCrAlY (where M is Ni, Co, or a combination of these), and a ceramic top layer based on yttria-stabilized zirconium dioxide (YSZ) [9,10].

The NiCrAlY (nickel–chromium–aluminum alloys) bonding layer forms a protective oxide layer, Al_2_O_3_, which effectively prevents further oxidation of the metal layer at high temperatures [11]. This improves thermal resistance and protects the metal from corrosion in aggressive environments, such as atmospheres with high chloride or sulfide content. Studies have confirmed that this material is highly resistant to oxidation and retains its mechanical properties at temperatures above 1000 °C [3]. Ceramic coating based on YSZ (stabilized zirconium oxide) plays the role of a thermal barrier due to its unique thermal insulation properties. Grains of tetragonal zirconia stabilized with yttrium oxide (Y_2_O_3_) prevent phase transformation and cracking under thermal cyclic stresses [12]. This ceramic coating has a low thermal conductivity (~2.5 W/m × K), which allows the temperature on the surface of the metal layer to be significantly reduced and protects it from overheating, which increases the durability of the system [6]. Studies [5,6] and scientists have shown that the use of ceramic layers stabilized with yttrium oxide significantly increases the corrosion resistance of coatings. This is due to the fact that the tetragonal phase ZrO_2_ stabilized by Y_2_O_3_ has increased resistance to phase transformations at high temperatures, which reduces the risk of crack formation and increases the durability of the coating under conditions of corrosion aggression [13].

However, an important problem in the use of such coatings is the mismatch of thermal expansion coefficients (TECs) between the metal and ceramic layers. During heating and cooling, metal expands and contracts faster than ceramics, which leads to the accumulation of thermal stresses under thermal cyclic loads and causes cracking of the coating [2]. Many studies have shown that this problem is the main cause of failure of thermal protective coatings. In [14], a mismatch in thermal expansion coefficients (TECs) between the metallic bonding layer and the ceramic top layer was identified as the main failure factor of thermal protective coatings (TBCs). This mismatch causes thermal stresses to build up under cyclic thermal loads, leading to cracking and peeling of the coating. This phenomenon is particularly common in plasma-sprayed coatings where high stresses occur at the interface between the NiCrAlY metal layer and the YSZ ceramic layer due to differences in the thermal expansion of the materials. Similar problems are also described in [15]. When the system is cooled, horizontal cracks appear in the ceramic layer and at the interface between the thermally grown oxide (TGO) and the ceramic layer (YSZ). These cracks are caused by the difference in thermal expansion between the different coating layers. The use of multilayered structures helps to reduce these stresses, but they cannot be completely avoided, especially at extreme temperatures.

Therefore, the reduction in thermal stresses in thermal protective coatings remains one of the key tasks in modern materials science. One of the promising directions for solving this problem is the development of layer gradient coatings, where the composition of materials changes gradually from the metal layer to the ceramic layer. This allows a uniform distribution of stresses and minimizes the likelihood of cracks at the boundaries between layers, as the difference in coefficients of thermal expansion (CTE) is smoothed out by the gradient transition. For example, Ref. [16] showed that such coatings have better resistance to thermal cyclic loading compared to conventional coating systems. Another study of YSZ/La_2_Ce_2_O_7_ double layers demonstrated that gradient coatings provide better thermal shock and hot corrosion resistance due to the gradual change in coating composition, which helps to reduce the risk of cracking at the layer interfaces [17]. Similar results were obtained in [14], where the use of NiCrAlY- and YSZ-based layered structures significantly improved the resistance of the coating to mechanical damage and thermal shock. Thus, the development of new approaches to reduce thermal stresses in thermal protective coatings is an urgent task.

One of the most promising methods for creating such gradient structures is detonation sputtering, which ensures tight adhesion of layers and minimizes the porosity of the coating [4]. Detonation spraying is a method of thermal spraying in which the coating is formed by heating and accelerating the powder material with the help of detonation products of the gas mixture. The process is carried out using special devices—detonation guns—in which a mixture of oxygen and fuel (usually acetylene) is ignited, creating a detonation wave. This wave accelerates the powder particles to supersonic speeds (up to 1000 m/s), which allows them to be deposited on the substrate surface, forming a dense, low-porosity coating with good adhesion. A key advantage of detonation spraying is the use of two dispensers for feeding different materials, which allows the creation of multilayered or layer gradient coatings with a smooth transition from the metal layer to the ceramic layer. This allows for a smooth change in coating composition from the metal layer to the ceramic layer, which helps to reduce thermal stresses by gradually changing the coefficients of thermal expansion (CTEs) between layers [14]. This technique provides a denser and more homogeneous coating and improves its wear resistance, corrosion resistance, and thermal shock resistance. Recent studies [4] have confirmed that detonation coatings show improved properties compared to plasma-sprayed coatings, especially under high temperatures and oxidizing environments.

An important aspect is not only the heat resistance but also the wear resistance of the coatings, especially under friction conditions [18]. A comparative study of different methods of applying heat-protective coatings [19] has shown that coatings obtained by detonation spraying have a higher resistance to mechanical wear compared to coatings obtained by plasma spraying. This is due to a denser structure and a smaller number of pores, which reduces the likelihood of penetration of aggressive substances and the occurrence of corrosion centers [16]. These data confirm that the detonation spraying method can be effective for creating coatings that are resistant not only to thermal loads but also to mechanical impacts, where high temperatures and mechanical loads act simultaneously. In addition, the heat resistance of the coating should be complemented by high corrosion resistance, as many gas turbines operate in aggressive environments containing sulfur, chlorides, and other corrosive active substances. Studies [20,21] and scientists have shown that the use of ceramic layers stabilized with yttrium oxide significantly increases the corrosion resistance of coatings. This is due to the fact that the tetragonal phase ZrO_2_ stabilized by Y_2_O_3_ has increased resistance to phase transformations at high temperatures, which reduces the risk of crack formation and increases the durability of the coating under conditions of corrosion aggression.

Thus, analyzing the latest research in the field of heat-protective coatings, we can conclude that the development of detonation spraying technologies and the creation of multilayer gradient structures are promising directions for improving the efficiency and durability of coatings at high temperatures and in aggressive environments. Special attention should be paid to the optimization of the microstructure of coatings and their thermal, mechanical, and corrosion characteristics, which will improve the performance properties of coatings and expand their application in power engineering and aircraft construction.

The purpose of this work was to study the structural phase, mechanical, and tribological characteristics of NiCrAlY- and YSZ-based layer gradient thermal protective coatings obtained by detonation spraying. The research involved the evaluation of their thermal and corrosion resistance, as well as the study of their behavior under conditions of high temperatures and aggressive media. Layer gradient thermal protective coatings formed by detonation spraying were created as follows. First, coatings were applied to the substrate (X6CrNiTi18-10 steel), which had been previously sandblasted, with a gradual change in the composition of the powders fed from the dosers into the detonation spraying barrel. For this purpose, two dispensers were used simultaneously: the ceramic powder feed was gradually increased, while the metal powder feed was gradually reduced. This approach made it possible to create a coating with a layer gradient structure. The technical result was the formation of a coating consisting of a lower metallic layer based on nickel (NiCrAlY) and an upper ceramic layer consisting of zirconium oxide powder (ZrO_2_) stabilized with yttrium oxide (Y_2_O_3_) on the surface of X6CrNiTi18-10 steel. The content of the ceramic component increased smoothly from the metal layer to the ceramic layer along the thickness of the coating. Coatings obtained in this way are characterized by a layer-gradient structure with a smooth transition (gradient) of chemical composition between the main coating zones. Gradual change in microstructure and physical properties of layers improves the performance characteristics of the heat-protective coatings. Such coatings have the necessary properties of the outer layers, which are exposed to the environment. In addition, the layer gradient structure reduces the difference in physical and mechanical characteristics between the coating material and the substrate, which in turn reduces the stress jump at the layer boundary that arises under loads.

## 2. Materials and Methods

In this work, X6CrNiTi18-10 stainless steel (ISO 15510:2014) of 20 × 15 × 3 mm was chosen as the substrate, and its surface was pre-treated by sandblasting. NiCrAlY powder was used for the metallic coating, and Metco 233B powder was used for the YSZ ceramic coating. The chemical composition of the powders and X6CrNiTi18-10 stainless steel are given in Table 1, Table 2 and Table 3, respectively.

**Figure 1:**
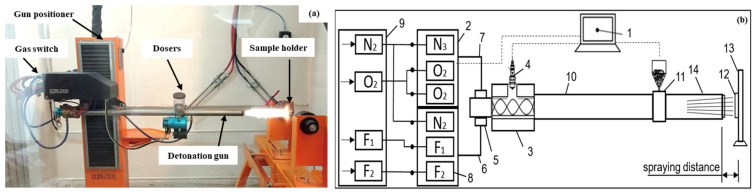
General view of the CCDS200 installation (**a**) and its process diagram (**b**): 1—control computer, 2—gas distributor, 3—mixing-ignition chamber, 4—spark plug, 5—barrel valve, 6—fuel line, 7—oxygen line, 8—gas valves, 9—gas supply unit, 10—breech of the barrel, 11—powder feeder (doser), 12—workpiece; 13—manipulator, 14—muzzle of the barrel.

The study was performed using the detonation spraying process using the CCDS2000 setup (LIH SB RAS, Novosibirsk, Russia) (Figure 1). For the application of the metallic NiCrAlY layer and the ceramic YSZ layer, two feeders were used, each delivering the corresponding powder. The metallic NiCrAlY powder was supplied through the first feeder, while the ceramic YSZ powder was supplied through the second. The gas mixtures used for spraying varied depending on the material being applied. A mixture of acetylene and oxygen (C_2_H_2_ + 0.97O_2_) was used to create a metallic layer of NiCrAlY with a barrel filling of 35%, while the coating dose was 2.3 μm per shot. A gas mixture including acetylene and oxygen (C_2_H_2_ + 2.52O_2_) was used to apply the ceramic layer of YSZ with a barrel filling of 61% and a dose of 1.7 μm per shot. The mechanism of gradient coating formation includes several stages. During detonation spraying, detonation of the gas mixture and powders occurs, which leads to the formation of high-temperature plasma and shock waves. These waves accelerate the powder particles to high speeds and heat them to a temperature sufficient for melting. High-energy particles impact the substrate surface, forming dense and uniform coating layers.

To obtain a gradient coating, two deposition regimes with different shot counts were developed. In the first regime (1D1), the number of NiCrAlY alloy shots was gradually reduced from 5 to 1 as the layers were deposited, while the number of YSZ shots was increased from 1 to 20. This created a smooth transition from the metallic to the ceramic layer, which provided a gradient coating structure. In the second regime (2D2), the number of NiCrAlY shots started from 10 and decreased to 2, while the number of YSZ shots was increased from 2 to 40. This regime also created a smooth transition from the metallic to the ceramic layer but with a higher number of YSZ shots, which resulted in a higher concentration of ceramics in the top coating layer. Spraying was performed at a distance of 200 mm from the substrate. The characteristics of the 1D1 samples and the 2D2 samples, as well as the coating production conditions, are presented in Table 4.

The crystallographic composition of the starting powder and coatings was determined using an X’Pert Pro X-ray diffractometer (Philips Corporation, Amsterdam, The Netherlands) with Cu-Ka radiation (λ = 1.54 Å). The phase composition was analyzed in the angle range of 20–90° following JCPDS cards: t’-ZrO_2_ (01-081-1545), m-ZrO_2_ (00-003-0515), NiCoCr (00-021-1271), and β-NiAl (00-044-11-87). The analyses were carried out using the Highscore Plus software version 3.0e.

The morphological features of the powder particles, as well as the surfaces and cross sections of the coatings, were characterized using TESCAN MIRA3 LMH scanning electron microscopy (SEM) (TESCAN, Brno, Czech Republic). Porosity was determined from cross sections of coatings in SEM images according to ASTM E2109 and using the Image J software version 1.54g [23].

The hardness and elastic modulus of the coatings were measured using the FISCHERSCOPE HM 2000 S system (Helmut Fischer GmbH, Sindelfingen, Germany) controlled by the WIN-HCU software version 7.1. This instrument is designed to evaluate the microhardness and other mechanical properties of materials in accordance with ISO14577. In all five tests, a holding time of 10 s was set at a load of 1 N. To evaluate the creep resistance of the coating under constant load conditions, the creep indentation rate (CIT) parameter was calculated. This parameter characterizes the change in indentation depth over time under constant load and is calculated as a percentage change in indentation depth relative to the holding time. The formula for calculating CIT is as follows (1):

(1) $$CIT=\frac{h\left(t\right)-h_{0}}{h_{0}}\cdot 100\%$$

where h(t) is the indentation depth at the moment in time, and h_0_ is the indentation depth immediately after applying a constant load.

Surface roughness (R_a_) was measured using a model 130 profilometer in accordance with the requirements of GOST 25142-82. To carry out measurements on layer gradient coatings, the speed of the profilometer measuring head was set at 0.25 mm/s, while the measuring scale was 500 µm, and the length of the scanning track was 10 mm.

Tribological tests were carried out on an Anton Paar TRb^3^ tribometer (Anton-Paar, Buchs, Switzerland), which complies with the international standards ASTM G 133-95 and ASTM G 99. The tests were carried out under dry friction conditions at room temperature with reciprocating motion. A tungsten carbide ball with a diameter of 6 mm was used as a contour body. The tests were carried out under a load of 10 N and a linear velocity of 10 cm/s; the wear radius was 5 mm, and the friction path reached 300 m. A SURTRONIC S-100 profilometer (London, UK) was used to evaluate the dependence of the wear depth on the scanning length. The depth and width of the wear profile were used to determine the degree of wear, and the wear rate was calculated using Formula (2) [24]:

(2) $$k=\frac{V}{F\times S}{mm}^{3}/N\times m$$

where k is the wear rate, V is the wear volume, F is the applied load, and S is the total sliding distance. Quantification of wear was carried out using Origin 2017 software version 9.4 using the ‘area under the curve’ calculation method.

Potentiodynamic polarization methods were used to evaluate the corrosion behavior of the samples. The measurements were carried out in a three-electrode electrolytic cell with separation of cathode and anode spaces. The experiments were carried out at 25 ± 10 °C in an atmosphere with free access to air and in NaCl solution with a concentration of 3.5 wt.%. A platinum electrode was used as an auxiliary electrode, and a choosier electrode (Ag/AgCl (1 M KCl)) was used as a reference electrode. Potentiodynamic polarization was started at −0.1 V and terminated at 0.1 V relative to the OCP, with a potential sweep of 0.5 mV/s. A CS300M potentiostat/galvanostat (Wuhan Corrtest Instruments Co., Ltd., located in Wuhan, China) was used for polarization and related measurements.

## 3. Results

**Figure 2:**
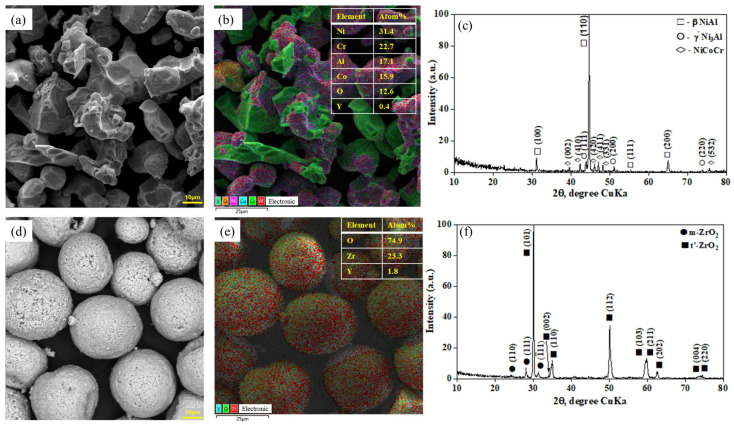
Microstructure of the powders and results of X-ray phase analysis of phase composition: (**a**–**c**) NiCrAlY powder; (**d**–**f**) YSZ powder.

Figure 2a,b, obtained with a scanning electron microscope, showed that the NiCrAlY powder mixture used as a metal layer contained irregularly shaped particles (shards). The particle size varied between 5 and 30 μm. This shape and particle size favored uniform distribution of the material during spraying, ensuring a tight fit in the coating structure. The phase composition of the powder included the NiAl intermetallic phase (β-phase), which accounted for 93.8% of the total volume, as well as minor amounts of γ’-Ni_3_Al (3.9%) and NiCoCr (2.3%) phases (Figure 2c).

The micrographs of the YSZ powder mixture, presented in Figure 2d,e, showed that the zirconium dioxide powder stabilized with yttrium oxide (YSZ) had spherical particles with a porous structure. The particle size varied from 15 to 40 μm. This structure contributed to the mechanical properties of the coating due to the high density and uniform distribution of the particles. X-ray phase analysis showed that the YSZ powder consisted of 89.52% tetragonal phase and 10.48% monoclinic phase (Figure 2f). During the detonation spraying process, disappearance of the monoclinic phase of ZrO_2_ was observed, indicating a significant improvement in the homogeneity of the coating structure.

**Figure 3:**
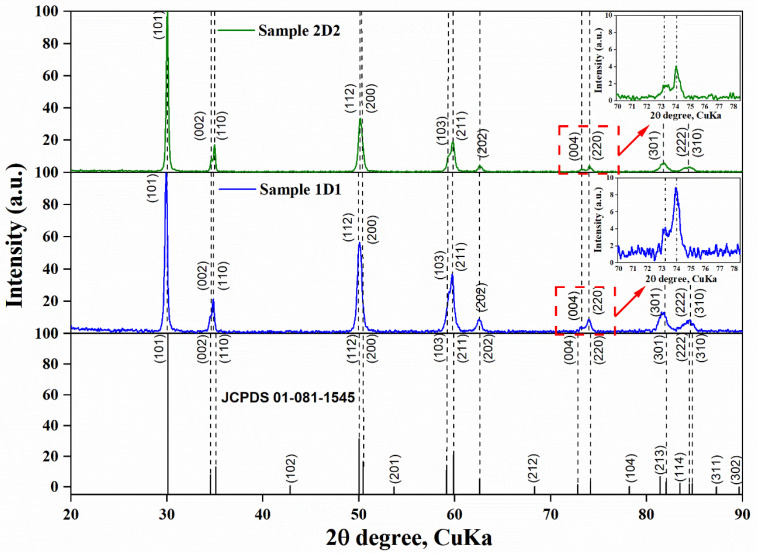
Diffraction spectra of layer gradient coatings.

X-ray phase analysis confirmed that during the detonation spraying process, there was a complete transformation of the monoclinic phase into the tetragonal phase (Figure 3). The phase transition of ZrO_2_ from monoclinic to tetragonal phase during detonation spraying was caused by high temperatures generated by the blast wave. The monoclinic phase, stable at room temperature, became unstable when heated above 2000 °C, and the crystal lattice was reconstructed into a more symmetrical tetragonal form. Rapid cooling of the particles after impact with the substrate prevented the material from returning to the monoclinic state, stabilizing the tetragonal phase. The tetragonal phase of ZrO_2_ was characterized by a complex microstructure including twins and antiphase boundaries, which increased the resistance of the material to crack propagation, in contrast to the monoclinic phase, which is prone to phase transformation and can cause coating failure.

However, due to similar lattice parameters, distinguishing between tetragonal and cubic zirconia structures can be difficult. As noted by Srinivasan et al. [24], the tetragonal structure can be distinguished from the cubic structure by the characteristic splitting of the Bragg peaks of the tetragonal phase, including pairs of peaks (002) and (200), (113) and (311), (004) and (400), and (006) and (600). These were observed in the 2θ regions of 72–76° and 120–132°. In contrast, the cubic phase had single peaks at all these positions. As can be seen in Figure 2, characteristic splitting of the Bragg peaks of the tetragonal phase was observed in the X-ray diffraction patterns of samples 1D1 and 2D2, indicating its predominance in the crystal structure of the sprayed YSZ top layers. Also, as shown in Figure 3, there were slight differences in the phase intensity of the samples. In particular, the phase reflections of the tetragonal phase in sample 1D1 had a higher intensity compared to sample 2D2. The weaker intensity of the diffraction maxima in sample 2D2 could be related to its higher porosity [25].

The tetragonal zirconium dioxide phase was divided into transformed (t) and non-transformed (t’) phases, which differed in the ratio of the cell parameters. For the t’-ZrO_2_ phase, the ratio c/a√2 tended to 1.010, whereas for the t-ZrO_2_ phase, it exceeded 1.010. According to the data presented in Table 4, in samples 1D1 and 2D2, the c/a√2 ratio was 1.008 and 1.009, respectively, indicating the predominance of the non-transformable t’-phase in their crystal structure. The lattice cell parameters and tetragonality value (c/a√2) of the samples are presented in Table 5.

**Figure 4:**
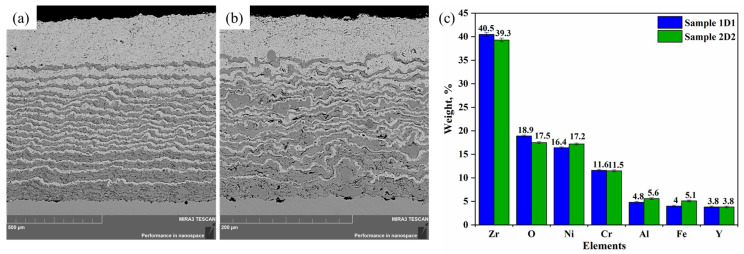
Cross-sectional BSE images of samples 1D1 (**a**) and 2D2 (**b**) with visible layered structure, as well as a histogram of the distribution of elements (Zr, O, Ni, Cr, Al, Fe, and Y) by their content in percentage (**c**).

Figure 4a,b show cross-sectional images of samples 1D1 and 2D2 obtained by backscattered electron (BSE) and elemental mapping results. All samples exhibited a layer gradient structure consisting of alternating layers of YSZ and NiCrAlY. The boundary between the coating layers can be clearly distinguished in the images, where the YSZ and NiCrAlY layers are visible as lightly and darkly colored layers, respectively. Elemental mapping of cross sections of the samples revealed the presence of the main elements of the coatings (Zr, O, Ni, Cr, Al, and Y) and the substrate (Fe), without impurities (Figure 4c).

The main difference between the samples was the coating thickness. Sample 1D1 had a significantly thicker coating of 963.67 ± 13.59 μm, while sample 2D2 had a coating thickness of 273.72 ± 1.26 μm. This was due to the different number of spraying cycles for each sample. The thicker coating in sample 1D1 was achieved by using a higher number of detonation spraying shots. Figure 4a,b also show that the NiCrAlY content gradually decreased towards the coating surface, whereas the YSZ concentration increased, indicating a smooth transition from the NiCrAlY layer to the YSZ layer. Due to the peculiarities of the process, the interfaces between the YSZ and NiCrAlY layers were rough but well distinguishable. This indicates the high-quality melting of YSZ and NiCrAlY powders in the detonation flame and their good adhesion to each other and to the stainless steel substrate. The bonding surface between the substrate and the coating was interdigitated, indicating high bond strength.

An important observation was the absence of visible cracks, defects, and signs of delamination on all samples. The coatings obtained by detonation spraying had a dense microstructure with low porosity. The pore volume fraction was 2.31% for sample 1D1 and 3.28% for sample 2D2. This can be attributed to the high particle velocity during the spraying process, which promotes significant plastic deformation of small particles upon collision with the substrate, reducing the gaps between them and minimizing gas retention within the coating. The black spots observed in the corner areas at the boundary between the coating and substrate were probably due to voids created by sandblasting.

**Figure 5:**
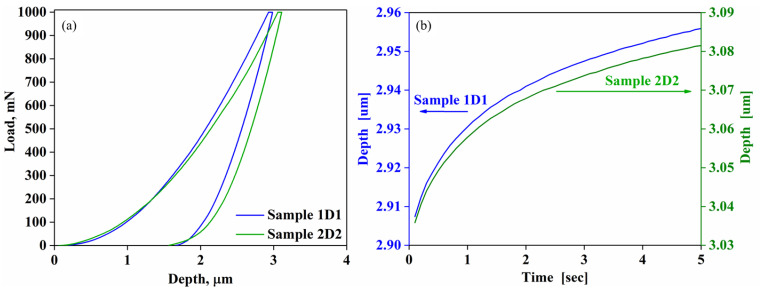
Hardness values as a function of penetration depth for two samples (**a**) and variation of indenter penetration depth over time at constant load (**b**).

The hardness values of detonation coatings as a function of penetration depth were obtained using the Martens method. During the tests, the indenter load and displacement on the surface were continuously measured, which made it possible to construct detailed curves of load versus penetration depth (Figure 5). Figure 5a shows such curves for two samples obtained with different numbers of detonation spraying shots. The mechanical characteristics of these samples are summarized in Table 6. As expected, the area between the loading and unloading curve and the maximum indenter penetration depth (h_max_) decreased with increasing surface roughness. This is due to the fact that a rougher surface provides greater resistance to indenter penetration. The surface roughness of sample 1D1 was 3.76 μm, which was significantly higher than that of sample 2D2, where it was 1.21 μm.

Despite the differences in the spraying process and roughness parameters, the data in Table 6 show that the hardness of the detonation coatings, Young’s modulus (E), plasticity index (H/E), elastic recovery (H^2^/E), and plastic deformation resistance (H^3^/E^2^) were almost the same. This fact indicates stability and high reproducibility of mechanical properties, which is probably explained by the homogeneous microstructure and density of the coatings formed by the detonation spraying method. The detonation spraying method, due to the high particle velocity and intense thermal impact, provides a dense coating structure with minimal porous zones. Such density minimizes the influence of process variables, such as the number of spraying cycles or roughness parameters. Uniform distribution of materials and homogeneity of the coating ensure that the mechanical properties of the coatings are maintained even when the application conditions or surface parameters change.

For creep analysis, the maximum load was kept constant for 5 s. Figure 4b shows the variation of the indenter penetration depth over time at constant load. It can be seen that the penetration depth of the indenter tip continued to increase when the load was held constant until the constant load ceased. This increase in penetration depth under constant load conditions is indicative of material creep.

Creep indentation rate (CIT) is an important parameter that characterizes the tendency of a material to deform under long-term loading. The creep rate values for different coatings were calculated and reported in [26]. In this study, the creep rate values calculated from the data presented in Figure 5b were 1.9% for the 1D1 detonation coating and 1.7% for the 2D2 coating. These results indicate similar creep behavior of the coatings with minor differences in the CIT values.

**Figure 6:**
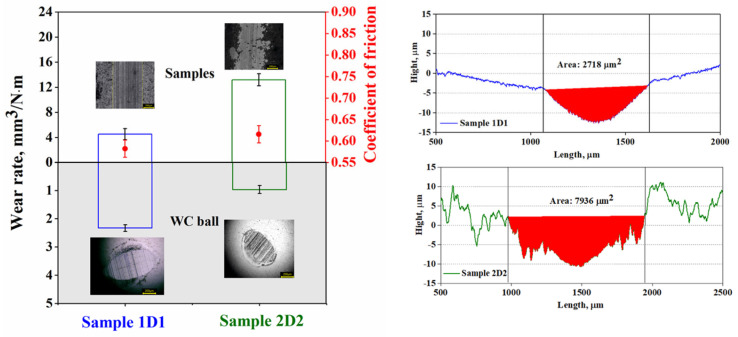
Average values of friction coefficient and wear rate for samples 1D1 and 2D2.

To evaluate the wear resistance of the coatings obtained by detonation spraying, sliding wear tests were carried out using a linear reciprocating tribometer at room temperature (300 K). The average values of friction coefficient and wear rate for samples 1D1 and 2D2 are shown in Figure 6. Sample 2D2 exhibited a higher friction coefficient (0.6161) compared to sample 1D1 (0.5826). In addition, the wear rate of sample 1D1 (4.53 × 10^−6^ mm^3^/(N × m)) was approximately three times lower than that of sample 2D2 (1.32 × 10^−5^ mm^3^/(N × m)), indicating greater wear resistance of sample 1D1.

The differences in wear resistance can be attributed to differences in the microstructural and surface properties of the coatings [27]. Sample 1D1 had a denser structure with less porosity compared to 2D2, which reduced the likelihood of cracks and defects that contribute to wear. The porosity of sample 2D2 increased stress concentration and crack initiation, resulting in a wider and deeper wear scar. The wear scar width of sample 2D2 was 635 μm, which was significantly greater than that of sample 1D1 (560 μm). The morphology of the worn surface of sample 2D2 indicated a greater tendency to delamination.

The morphology of the worn surfaces of the WC counter body was analyzed using optical microscopy (Figure 6). The analysis showed that the counter body used in the testing of sample 1D1 lost 1.6 times more wear area compared to the counter body applied to coating 2D2. This difference correlates well with the wear rate of the coatings and indicates a significant difference in their wear resistance, which can be attributed to differences in microstructure and surface roughness [18]. The worn morphology of the counter body had an approximately ellipsoidal shape, indicating a uniform wear distribution over the entire contact area between the counter body and the coating. Numerous scratches were observed on the surface of the WC balls, resulting from the intense abrasive interaction between the coating and the counter body, which resulted in the removal of material from the coating surface. One of the factors affecting wear resistance is the surface roughness of the coatings. The higher surface roughness of sample 1D1 may have contributed to more wear of the WC counter body, as irregularities in the coating surface increase the mechanical impact on the counter body.

**Figure 7:**
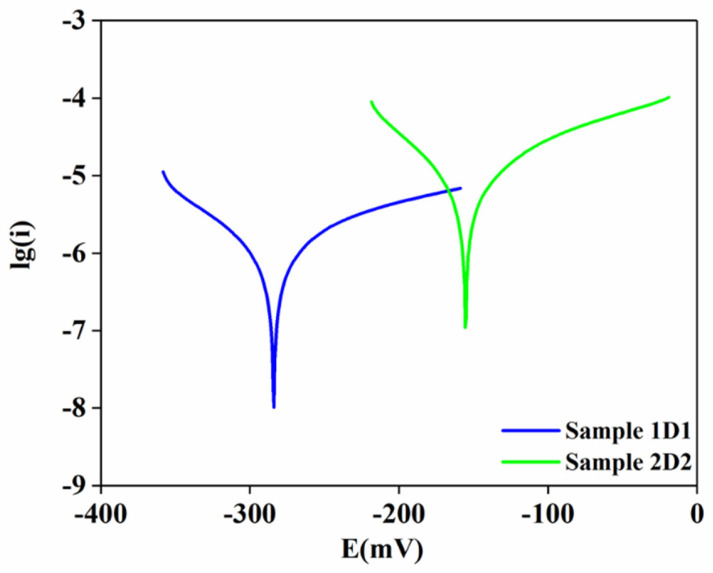
Potentiodynamic polarization curves of samples 1D1 and 2D2 in a 3.5% NaCl solution recorded for 30 min at room temperature.

Figure 7 shows the potentiodynamic polarization curves of samples 1D1 and 2D2 in a 3.5% NaCl solution recorded for 30 min at room temperature. As can be seen from the curves, both coatings exhibited active behavior. The corrosion potential shifted to more negative values for samples 1D1 (−280.3 mV) and 2D2 (−156.2 mV) in sodium chloride solution. It can be seen that the potential values of both coatings remained stable throughout the test. A number of electrochemical parameters, such as corrosion potential (E_corr_), corrosion current density (I_corr_), anodic (βa) and cathodic (βc) slope, corrosion rate (r_corr_), and polarization resistance (Rp), were calculated based on nonlinear fitting of Tafel plots. These parameters are summarized in Table 7.

As can be seen in Figure 7, sample 2D2 had a more positive corrosion potential than sample 1D1, that is, sample 2D2, compared to coating 1D1, showed a positive shift to −156.22 V. However, higher corrosion potential does not always indicate better corrosion resistance [28]. It is important to consider the corrosion current density (I_corr_), which is a key parameter to evaluate the corrosion reaction kinetics. Sample 2D2 showed a higher corrosion current density ((1.20 ± 0.15) × 10^−5^ A/cm^2^) compared to sample 1D1 ((2.19 ± 0.54) × 10^−6^ A/cm^2^). The lower value of I_corr_ indicates higher corrosion resistance. This indicates higher corrosion activity of the 2D2 coating. The lower corrosion current density of sample 1D1 indicates lower corrosion activity of the coating. This confirms the decrease in corrosion rate by more than ten times after sealing treatment. The corrosion rate for sample 2D2 was 0.14 mm/yr, which was higher than that of sample 1D1 (0.03 mm/yr). This indicates that sample 2D2 would degrade faster in the corrosive environment compared to sample 1D1.

The anodic and cathodic slopes (βa and βc) are also important parameters to understand the behavior of the coatings. For sample 2D2, the values of βa and βc were 143.31 and 87.53 mV, respectively, while they were 231.61 and 114.31 mV for sample 1D1. The higher values of these parameters indicate a more pronounced anodic and cathodic behavior in the 1D1 coating, which may indicate a more stable passivation layer. Polarization resistance (Rp) is an important parameter that reflects the ability of the coating to resist corrosion. The polarization resistance for sample 1D1 (17,400.7 Ω × cm^2^) was significantly higher than that of sample 2D2 (2060.3 Ω × cm^2^), indicating better corrosion protection for sample 2D2.

**Figure 8:**
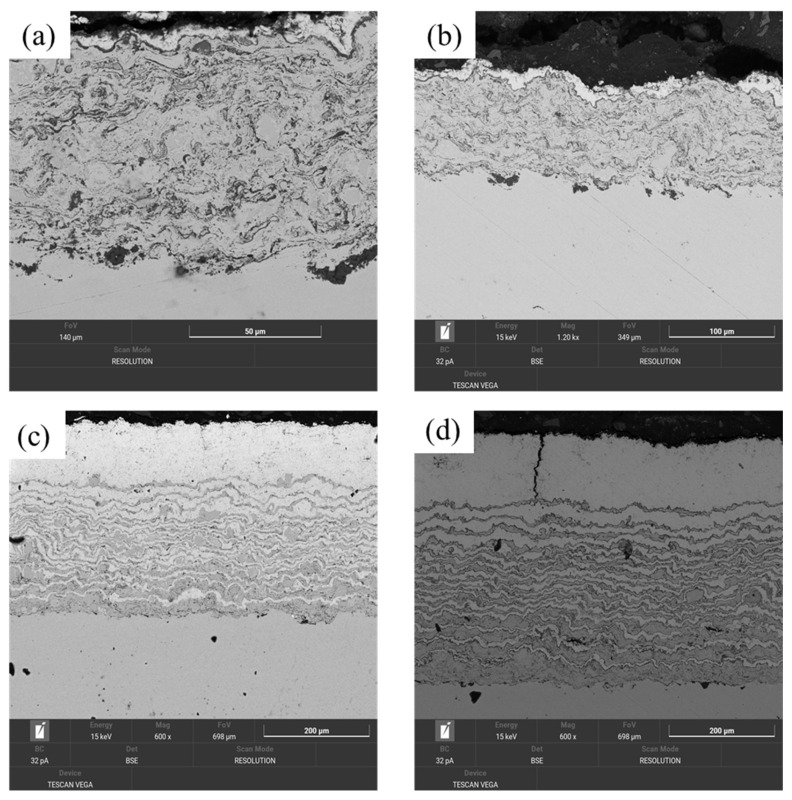
SEM images of cross sections of samples 1D1 (**a**,**b**) and 2D2 (**c**,**d**) after annealing at 1000 °C with subsequent cooling in air (**a**,**c**) and in vacuum (**b**,**d**).

Thermal shock tests were carried out at 1000 °C, followed by cooling in both air and vacuum. These thermal tests allowed us to evaluate the resistance of the coatings to thermal shock and microstructure changes depending on the cooling conditions. SEM images of cross sections of samples 1D1 and 2D2 after annealing at 1000 °C with subsequent cooling in air and vacuum, presented in Figure 8, showed three types of failure: formation of a single deep crack (Figure 8d), formation of a shallow crack network (Figure 8c), and peeling of the thermal protection coating (Figure 8a,c). These failures can be explained by the combined effects of thermal processes, element diffusion, and stress relaxation at interfacial interfaces, which resulted in significant changes in the coating structure. A single deep crack (Figure 8d) was observed in 2D2 samples after vacuum annealing. This phenomenon is probably due to the non-uniform stress distribution caused by the differences in thermal expansion coefficients between the coating layers. At the same time, a shallow crack network (Figure 8c) appeared in 2D2 samples after air cooling. This failure may be the result of localized stresses that contributed to the formation of multiple cracks in the top coating layer. The coating delamination (Figure 8a,b) observed in samples 1D1 after annealing under both vacuum and air cooling may be a consequence of the accumulation of tensile stresses at the interfacial interfaces. These stresses, along with thermal degradation of the bonding between the layers, resulted in a loss of coating integrity. The thermal shock resistance of a thermal protection coating also depends on coating characteristics such as cracking, thickness, density, surface roughness of the bonding layer, and residual stresses after detonation spraying. Increasing the layer thickness reduces the thermal shock resistance, as demonstrated in sample 1D1, where delamination of the coating from the ceramic layer and at the top layer/binder interface occurred.

It is important to note that air cooling and vacuum cooling have different effects on the heat treatment behavior of the coatings. Air cooling causes a sharper temperature gradient, which favors the formation of a shallow crack network in the top layer of the coating. On the other hand, vacuum cooling may favor a more uniform temperature distribution, which increases the likelihood of deep single cracks, as observed in 2D2 samples. In addition, the failure of coatings can also be due to diffusion of elements between layers during annealing. These diffusion processes can change the chemical composition at the interfaces, weakening the bond between the layers and increasing the likelihood of coating delamination.

**Figure 9:**
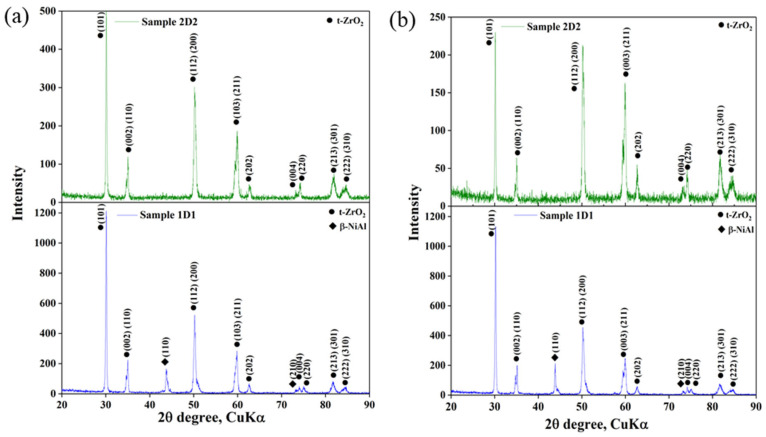
Diffraction patterns of coatings after annealing: (**a**) air cooling; (**b**) vacuum cooling.

In particular, after thermal shock, the YSZ/NiCrAlY layer in coating 1D1 had completely detached from the NiCrAlY bonding layer in some areas, as shown in Figure 8. To determine the phases present in the top coating at the time of fracture, X-ray diffraction analysis of the detached top layer was performed. As shown in Figure 9, the analysis results revealed the presence of very small nickel alloy peaks.

Table 8 presents the results of phase analysis and lattice parameter determination of the thermally annealed top layers of the coatings. X-ray diffraction analysis revealed significant changes in the phase composition and lattice parameters of the coatings after heat treatment.

In 1D1 samples cooled in air, the tetragonal ZrO_2_ (t-ZrO_2_) phase predominated, accounting for 98.2% of the total coating volume, while the presence of β-NiAl was insignificant and amounted to only 1.8%. This indicates the stability of the main phase under these cooling conditions, while β-NiAl had minimal effect on the coating structure. For vacuum-cooled 1D1 samples, an increase in the fraction of β-NiAl to 7.5% and a decrease in the fraction of t’-ZrO_2_ to 92.5% were observed. These changes in the phase composition indicate the influence of the cooling environment on the thermal behavior of the coating. The air-cooled 2D2 samples were entirely composed of t-ZrO_2_, indicating the retention of this phase during faster cooling. In 2D2 samples cooled in vacuum, the entire top layer was represented by t’-ZrO_2_, confirming the phase transformation under uniform cooling conditions.

## 4. Conclusions

The following conclusions can be drawn from the data obtained:

(1) X-ray phase analysis of the coatings confirmed that in the process of detonation sputtering, there was a complete transformation of ZrO_2_ from the monoclinic phase to the tetragonal phase due to the effect of high temperatures arising from the impact of the blast wave. This is due to the fact that the monoclinic phase, which is stable at room temperature, becomes unstable when heated above 2000 °C and transforms into a more symmetric tetragonal form, which then becomes stable due to the rapid cooling of the particles after impact with the substrate. Bragg peak analysis confirmed the predominance of the tetragonal phase in samples 1D1 and 2D2, with more intense phase reflections observed in sample 1D1, which may indicate a denser coating structure compared to sample 2D2, which was more porous. Additionally, the crystal structure of samples 1D1 and 2D2 was found to be dominated by a non-transformable e’-phase of tetragonal structure, as evidenced by the ratios of cell parameters c/a√2 being 1.008 and 1.009, respectively. This also confirms the stabilization of the material as a t’-phase, which improves the overall mechanical properties of the coating and its durability in high temperature applications.
(2) Hardness tests of the detonation coatings by the Martens method showed that despite the differences in surface roughness (3.76 µm for sample 1D1 and 1.21 µm for sample 2D2), mechanical characteristics such as hardness, Young’s modulus, plasticity index, and plastic deformation resistance were almost the same for both samples. This indicates the stability and reproducibility of the mechanical properties provided by the detonation spraying method, which is associated with the homogeneous microstructure and dense structure of the coatings. The detonation spraying method provides high density and minimal porous zones, which reduces the influence of technological variables such as the number of spraying cycles or roughness parameters. Creep analysis showed similar behavior of the samples, where the creep rate was 1.9% for sample 1D1 and 1.7% for sample 2D2, indicating small differences in the long-term load resistance of the coatings.
(3) Sliding wear tests showed that coating 1D1 had better wear resistance compared to coating 2D2, as evidenced by a lower coefficient of friction (0.5826) and a significantly lower wear rate (4.53 × 10^−6^ mm^3^/(N × m) versus 1.32 × 10^−5^ mm^3^/(N × m) for 2D2). This can be explained by the denser structure and lower porosity of the 1D1 coating, which reduced stress concentration and the risk of crack formation, while the more porous structure of the 2D2 coating favored more wear. Differences in wear resistance were also evident in the width of the wear marks, which was smaller in sample 1D1 (560 µm vs. 635 µm in 2D2) and in the wear pattern of the counter body, indicating greater mechanical resistance of coating 1D1.
(4) Electrochemical tests on the corrosion resistance of coatings 1D1 and 2D2 in a 3.5% NaCl solution showed that, despite the more positive corrosion potential of 2D2 (−156.2 mV vs. −280.3 mV for 1D1), its corrosion resistance was lower. This was confirmed by the higher corrosion current density of sample 2D2 (1.20 × 10^−5^ A/cm^2^), indicating a more active corrosion reaction compared to sample 1D1 (2.19 × 10^−6^ A/cm^2^). The corrosion rate of sample 2D2 was also higher (0.14 mm/year versus 0.03 mm/year for 1D1), indicating a faster degradation of the 2D2 coating in the corrosive environment. The high polarization resistance of sample 1D1 (17,400.7 Ω × cm^2^) compared to 2D2 (2060.3 Ω × cm^2^) confirmed the better corrosion protection of coating 1D1.
(5) Thermal tests showed that the thermal shock resistance of thermal protection coatings is significantly dependent on the cooling conditions and coating thickness. Sample 2D2 exhibited a single deep crack formation after vacuum annealing due to uneven stress distribution, whereas a shallow crack network appeared when cooled in air. Sample 1D1 was more prone to coating peeling, especially at the interlayer boundaries, due to the increased coating thickness and stress accumulation. In addition, diffusion of elements during heat treatment also affected the weakening of interfacial boundaries and increased the probability of coating failure. X-ray diffraction analysis showed that air-cooled samples 1D1 were dominated by the tetragonal ZrO_2_ phase, whereas an increase in the proportion of β-NiAl was observed during vacuum cooling, indicating the influence of the cooling environment on the phase composition of the coating. In 2D2 samples cooled in air, the t-ZrO_2_ phase was preserved, while in samples cooled in vacuum, the phase transformation into t’-ZrO_2_ occurred, which also indicates the influence of uniform cooling on the coating structure.

In this way, a layer gradient thermal protection material was created by detonation spraying, in which metal NiCrAlY layers and ceramic YSZ layers alternated smoothly. Analyzing the results of the study of layer gradient coatings, we can conclude that the development of detonation spraying technologies and the creation of layer gradient structures are promising directions for improving the efficiency and durability of coatings at high temperatures and aggressive environments. Optimization of the microstructure as well as improvement of thermal, mechanical, and corrosion characteristics of coatings are the key factors for increasing their operational properties. This will help to expand the application of such coatings in various fields, including power engineering and aerospace, where materials with high resistance to extreme conditions are required.

## Figures and Tables

**Table 1:** Chemical composition of X6CrNiTi18-10 stainless steel (wt.%).

C	Si	Mn	P	S	Cr	Mo	Ni	V	Ti	Cu	Fe
<0.08	<1.0	<2	<0.045	<0.015	17–19	<0.5	9–11	<0.2	<0.8	<0.3	other

**Table 2:** Chemical composition of NiCrAlY powder (wt.%).

Fe	Cr	Ni	Co	Mo	Al	Y, Si, Nb, C
<0.3	20	base	20	-	13	0.01–0.15

**Table 3:** Chemical composition of Metco 233B powder (wt.%).

ZrO_2_	Y_2_O_3_	SiO_2_	Al_2_O_3_	Fe_2_O_3_	TiO_2_	Other Oxides
Bal.	7.0–9.0	0.5	0.2	0.2	0.2	0.8

**Table 4:** Spraying regimes for NiCrAlY/ZrO_2_–Y_2_O_3_ (YSZ) coatings [22].

Layer	Material	Regime I (1D1)		Regime II (2D2)	
		Doser 1 (NiCrAlY)	Doser 2 (YSZ)	Doser 1 (NiCrAlY)	Doser 2 (YSZ)
		Number of Shots			
Substrate	12Kh18N10T	-	-	-	-
Layer 1	NiCrAlY/YSZ	5	1	10	2
Layer 2	YSZ/NiCrAlY	3	1	6	2
Layer 3	NiCrAlY/YSZ	2	1	4	2
Layer 4	YSZ/NiCrAlY	2	1	4	2
Layer 5	NiCrAlY/YSZ	1	1	2	2
Layer 6	YSZ/NiCrAlY	1	1	2	2
Layer 7	NiCrAlY/YSZ	1	1	2	2
Layer 8	YSZ/NiCrAlY	1	1	2	2
Layer 9	NiCrAlY/YSZ	1	1	2	2
Layer 10	YSZ/NiCrAlY	1	1	2	2
Layer 11	NiCrAlY/YSZ	1	1	2	2
Layer 12	YSZ/NiCrAlY	1	2	2	4
Layer 13	NiCrAlY/YSZ	1	2	2	4
Layer 14	YSZ/NiCrAlY	1	3	2	6
Layer 15	NiCrAlY/YSZ	1	20	2	40

**Table 5:** Lattice cell parameters and tetragonality value (c/a√2) of samples.

Samples	Detected Phase	Lattice Parameters, Å	c/a√2
1D1	t’-ZrO_2_	a = 3.6082	1.008
		c = 5.1436	
2D2	t’-ZrO_2_	a = 3.6117	1.009
		c = 5.1539	

**Table 6:** Mechanical characteristics of detonation layer gradient coatings.

Samples	HM, GPa	E, GPa	H/E	H^2^/E	H^3^/E^2^	h_max_, µm
1D1	4.359 ± 599	112 ± 15	0.039	0.17	0.000059	2.95589
2D2	3.976 ± 387	109 ± 13	0.036	0.15	0.000049	3.08149

**Table 7:** Electrochemical corrosion parameters of samples 1D1 and 2D2.

Samples	E_corr_ (mV)	I_corr_ (A/cm^2^)	β_c_ (mV)	β_a_ (mV)	r_corr_ (mm/yr)	Rp (Ω × cm^2^)
1D1	–280.25 ± 0.01	(2.19 ± 0.54)·10^−6^	114.31 ± 13.21	231.61 ± 8.31	0.03 ± 0.01	17,400.7 ± 3537.2
2D2	–156.22 ± 0.32	(1.20 ± 0.15)·10^−5^	87.53 ± 10.21	143.31 ± 9.72	0.14 ± 0.02	2060.3 ± 459.6

**Table 8:** Phases and lattice parameters found in the top layers of the coatings.

Samples	Detected Phase	Phase Content, Mas. %	Lattice Parameters, Å	c/a√2
1D1—air cooling	β-NiAl	1.8%	a = 2.8753	-
	t-ZrO_2_	98.2%	a = 3.5981	1.017
			c = 5.1781	
1D1—vacuum cooling	β-NiAl	7.5%	a = 2.8979	-
	t’-ZrO_2_	92.5%	a = 3.5962	1.009
			c = 5.1336	
2D2—air cooling	t-ZrO_2_	100%	a = 3.1603	1.153
			c = 5.1516	
2D2—vacuum cooling	t’-ZrO_2_	100%	a = 3.6291	0.997
			c = 5.1063	

## Data Availability

The original contributions presented in the study are included in the article, further inquiries can be directed to the corresponding author.

## References

[1] Bursich S., Morelli S., Bolelli G., Cavazzini G., Rossi E., Mecca F.G., Petruzzi S., Bemporad E., Lusvarghi L. (2024). The Effect of Ceramic YSZ Powder Morphology on Coating Performance for Industrial TBCs. Surf. Coat. Technol..

[2] Cheng T., Dong Y., Ma L., Wu Z., Wang J., Ma X., Wang Z., Dai S. (2024). Experiment and Numerical Simulation on Thermal Cycling Performance of YSZ-Based Sealing Coatings with “Brick-Mud” Layered Structure. Coatings.

[3] Erdogan G. (2023). Understanding the Sintering Behavior and Its Effect on the Thermal Conductivity of YSZ Coatings for Gas Turbine Applications. J. Aviat..

[4] Almomani M.A., Mahafdeh Q., Almomani A., Bataineh O. (2024). Selecting the Best Thermal Barrier Coating Material for Hot Sections in Gas Turbine Using AHP and TOPSIS Multi-Criteria Decision Making Techniques. Int. J. Interact. Des. Manuf..

[5] Ashofteh A., Rajabzadeh M. (2024). Advances in Thermal Barrier Coatings Modeling, Simulation, and Analysis: A Review. J. Eur. Ceram. Soc..

[6] Lokachari S., Leng K., Rincon Romero A., Curry N., Brewster G., Norton A., Hussain T. (2024). Processing–Microstructure–Properties of Columns in Thermal Barrier Coatings: A Study of Thermo-Chemico-Mechanical Durability. ACS Appl. Mater. Interfaces.

[7] Tao Q., Wang Y., Zheng Y. (2024). Fatigue Behaviour and Life Prediction of YSZ Thermal Barrier Coatings at Elevated Temperature under Cyclic Loads. Coatings.

[8] Ikpe A.E., Ekanem I.I., Ikpe E.O. (2024). A Comprehensive Study on Thermal Barrier Coating Techniques in High-Temperature Applications. Mech. Technol. Eng. Insights.

[9] Franco D., Vargas F., López E., Ageorges H. (2024). Wear Behavior at High Temperature of ZrO_2_–Y_2_O_3_ (YSZ) Plasma-Sprayed Coatings. J. Mater. Sci..

[10] Bie Y., Ren H., Bui T.Q., Madenci E., Rabczuk T., Wei Y. (2024). Dual-Horizon Peridynamics Modeling of Coupled Chemo-Mechanical-Damage for Interface Oxidation-Induced Cracking in Thermal Barrier Coatings. Comput. Methods Appl. Mech. Eng..

[11] Gautam S.S., Singh R., Vibhuti A.S., Sangwan G., Mahanta T.K., Gobinath N., Feroskhan M. (2022). Thermal Barrier Coatings for Internal Combustion Engines: A Review. Mater. Today Proc..

[12] Singh R.G., Lyons K.M., Waddell J.N., Li K.C. (2022). Effect of Thermocycling on the Mechanical Properties, Inorganic Particle Release and Low Temperature Degradation of Glazed High Translucent Monolithic 3Y-TZP Dental Restorations. J. Mech. Behav. Biomed. Mater..

[13] Warcholinski B., Gilewicz A., Lupicka O., Rochowicz J., Sayenko S., Svitlychnyi Y., Zykova A. (2014). Effect of Zirconia Stabilized by Ittria Additions on the Structure and Mechanical Properties of Alumina Based Ceramics. Funct. Mater..

[14] Hu Z.-C., Liu B., Wang L., Cui Y.-H., Wang Y.-W., Ma Y.-D., Sun W.-W., Yang Y. (2020). Research Progress of Failure Mechanism of Thermal Barrier Coatings at High Temperature via Finite Element Method. Coatings.

[15] Savitha U., Reddy G.J., Singh V., Gokhale A.A., Sundararaman M. (2020). Additive Laser Deposition of Compositionally Graded NiCrAlY-YSZ Multi-Materials on IN625-NiCrAlY Substrate. Mater. Charact..

[16] Amer M., Abdelgawad A., Curry N., Arshad M., Hayat Q., Janik V., Nottingham J., Bai M. (2024). SEM-Guided Finite Element Simulation of Thermal Stresses in Multilayered Suspension Plasma-Sprayed TBCs. Coatings.

[17] Pakseresht A., Ariharan S., Sekar A., Parchovianský M. (2024). Investigating Hot Corrosion, CMAS, and Thermal Shock Behaviour of Double-Layer YSZ/La_2_Ce_2_O_7_ + YSZ Thermal Barrier Coatings. J. Therm. Spray Technol..

[18] Tian J., Qi X., Xian G. (2024). Effect of Hygrothermal Aging on the Friction Behavior and Wear Mechanism of the Multi-Filler Reinforced Epoxy Composites for Coated Steel. J. Mater. Res. Technol..

[19] Sezavar A., Sajjadi S.A., Babakhani A., Peng R.L. (2019). Thermal Cyclic Fatigue Behavior of Nanostructured YSZ/NiCrAlY Compositionally Graded Thermal Barrier Coatings. Oxid. Met..

[20] Rakhadilov B., Sulyubayeva L., Maulet M., Sagdoldina Z., Buitkenov D., Issova A. (2024). Investigation of High-Temperature Oxidation of Homogeneous and Gradient Ni-Cr-Al Coatings Obtained by Detonation Spraying. Coatings.

[21] Scotson D., Paksoy A.H., Xiao P. (2024). Characterisation Techniques for Investigating TBC and EBC Failure: A Review. Front. Ceram..

[22] Buitkenov D., Nabioldina A., Raisov N. (2024). Development of Method for Applying Multilayer Gradient Thermal Protective Coatings Using Detonation Spraying. Coatings.

[23] Sablina T.Y., Sevostyanova I., Shlyakhova G. (2022). Influence of the Grain Size of ZrO_2_ (Y_2_O_3_) Ceramics on the Hardness, Fracture Toughness and Formation of a Transformation-Deformation Relief in the Indentation Zone. Russ. Phys. J..

[24] Srinivasan R., De Angelis R.J., Ice G., Davis B.H. (1991). Identification of Tetragonal and Cubic Structures of Zirconia Using Synchrotron X-Radiation Source. J. Mater. Res..

[25] Baizhan D., Sagdoldina Z., Buitkenov D., Kambarov Y., Nabioldina A., Zhumabekova V., Bektasova G. (2023). Study of the Structural-Phase State of Hydroxyapatite Coatings Obtained by Detonation Spraying at Different O_2_/C_2_H_2_ Ratios. Crystals.

[26] Alvarado-Rivera J., Muñoz-Saldaña J., Castro-Beltrán A., Quintero-Armenta J.M., Almaral-Sánchez J.L., Ramírez-Bon R. (2007). Hardness and Wearing Properties of SiO_2_–PMMA Hybrid Coatings Reinforced with Al_2_O_3_ Nanowhiskers. Phys. Status Solidi.

[27] Kengesbekov A., Rakhadilov B., Sagdoldina Z., Buitkenov D., Dosymov Y., Kylyshkanov M. (2022). Improving the Efficiency of Air Plasma Spraying of Titanium Nitride Powder. Coatings.

[28] Rakhadilov B., Muktanova N., Kakimzhanov D., Satbayeva Z., Kassenova L., Magazov N. (2024). Investigation of the Influence of Powder Fraction on Tribological and Corrosion Characteristics of 86WC-10Co-4Cr Coating Obtained by HVOF Method. Coatings.

